# Macrophages rely on extracellular serine to suppress aberrant cytokine production

**DOI:** 10.1038/s41598-021-90086-w

**Published:** 2021-05-27

**Authors:** Kento Kurita, Hiroya Ohta, Ibuki Shirakawa, Miyako Tanaka, Yasuyuki Kitaura, Yorihiro Iwasaki, Takashi Matsuzaka, Hitoshi Shimano, Seiichiro Aoe, Hiroshi Arima, Yoshihiro Ogawa, Ayaka Ito, Takayoshi Suganami

**Affiliations:** 1 Department of Molecular Medicine and Metabolism, Research Institute of Environmental Medicine, Nagoya University, Nagoya, Aichi Japan; 2 Department of Endocrinology and Diabetes, Nagoya University Graduate School of Medicine, Nagoya, Aichi Japan; 3 Department of Immunometabolism, Nagoya University Graduate School of Medicine, Nagoya, Aichi Japan; 4 Laboratory Nutritional Biochemistry, Department of Applied Biosciences, Graduate School of Bioagricultural Sciences, Nagoya University, Nagoya, Aichi Japan; 5 Center for Diabetes and Endocrinology, The Tazuke Kofukai Medical Research Institute Kitano Hospital, Osaka, Osaka Japan; 6 Department of Endocrinology and Metabolism, Faculty of Medicine, University of Tsukuba, Tsukuba, Ibaraki Japan; 7 Department of Home Economics, Otsuma Women’s University, Tokyo, Japan; 8 Department of Medicine and Bioregulatory Science, Graduate School of Medical Sciences, Kyushu University, Fukuoka, Fukuoka Japan; 9 Department of Pharmacology, Faculty of Pharmaceutical Sciences, Tokushima Bunri University, Tokushima, Japan

**Keywords:** Monocytes and macrophages, Cytokines, Inflammation, Nutrition

## Abstract

A growing body of evidence indicates that cellular metabolism is involved in immune cell functions, including cytokine production. Serine is a nutritionally non-essential amino acid that can be generated by de novo synthesis and conversion from glycine. Serine contributes to various cellular responses, but the role in inflammatory responses remains poorly understood. Here, we show that macrophages rely on extracellular serine to suppress aberrant cytokine production. Depleting serine from the culture media reduced the cellular serine content in macrophages markedly, suggesting that macrophages depend largely on extracellular serine rather than cellular synthesis. Under serine deprivation, macrophages stimulated with lipopolysaccharide showed aberrant cytokine expression patterns, including a marked reduction of anti-inflammatory interleukin-10 expression and sustained expression of interleukine-6. Transcriptomic and metabolomics analyses revealed that serine deprivation causes mitochondrial dysfunction: reduction in the pyruvate content, the NADH/NAD^+^ ratio, the oxygen consumption rate, and the mitochondrial production of reactive oxygen species (ROS). We also found the role of mitochondrial ROS in appropriate cytokine production. Thus, our results indicate that cytokine production in macrophages is tightly regulated by the nutritional microenvironment.

## Introduction

Macrophages play multifunctional roles in immune responses, for which cytokine production is an essential process during infection and tissue injury. Cytokine production in macrophages is tightly regulated in response to the microenvironment at the site of inflammation, in order to avoid persistent inflammation and subsequent tissue damage^1^. In addition to a variety of pathogenic stimuli, an emerging field called “immunometabolism” proposes the involvement of cellular metabolism such as glycolysis and fatty acid oxidation in the regulation of immune cell function, including cytokine production^2,3^. In this context, we and others have previously demonstrated that saturated fatty acids induce proinflammatory cytokine expression in macrophages by activating signaling pathways downstream of toll-like receptor 4 (TLR4) and endoplasmic reticulum stress^4–6^. Our data also indicated that saturated fatty acids upregulate the expression of genes related to serine metabolism^4^, suggesting a complex link between the metabolic pathways under inflammatory conditions. Although recent studies have indicated the role of essential amino acids such as tryptophan and histidine in immune cell function^7^, the role of non-essential amino acids such as serine in macrophage cytokine production remains poorly understood.

Serine is considered a nutritionally non-essential amino acid, because it can be synthesized de novo from 3-phosphoglycerate, an intermediate in glycolysis, by phosphoglycerate dehydrogenase (PHGDH)^8^. Serine contributes to various cellular responses, such as nucleotide synthesis, methylation reactions, and antioxidant defense. Indeed, cellular serine is indispensable for cell proliferation in immune cells and tumors^9–11^. Cellular serine is also provided by uptake of extracellular serine via neutral amino acid transporters and by serine hydroxymethyltransferase (SHMT)–mediated conversion from glycine^8^. In this regard, certain cell types such as neurons and tumor cells cannot synthesize sufficient serine and thus are dependent on exogenous serine provided by surrounding supporting cells or dietary intake of serine^12–14^. Recently, several lines of evidence indicated that supplementation of serine in diet or injection of serine effectively attenuates proinflammatory cytokine expression in animal models of bacterial infection and tissue injury^15–18^. In stark contrast, serine deficiency is known to inhibit interleukin-1β (*Il1β*) expression in macrophages through glutathione and one-carbon metabolism^19,20^. Thus, how serine regulates inflammatory responses remains controversial. It is also important to elucidate serine’s mechanism of action, since cellular serine is derived from more than one source.

Here, we show that depleting serine from the culture media markedly reduces serine levels in macrophages, indicating that the cellular serine levels are not compensated by cellular synthesis or conversion from glycine. When deprived of serine, macrophages stimulated with lipopolysaccharide (LPS) exhibit aberrant cytokine expression patterns, among which expression of anti-inflammatory interleukin-10 (IL10) is markedly inhibited, thereby sustaining proinflammatory cytokine expression. A combination of transcriptomic and metabolomics analyses revealed that serine deprivation reduces cellular pyruvate content, its transport into mitochondria, and the NADH/NAD^+^ ratio. We also found that the mitochondrial dysfunction results in impaired production of reactive oxygen species (ROS) in mitochondria and causes aberrant cytokine expression. This study demonstrates that extracellular serine is required for LPS-induced IL10 production, thereby suppressing sustained proinflammatory cytokine expression.

## Results

### Macrophages under inflammatory conditions require exogenous serine to maintain intracellular serine levels

**Figure 1:**
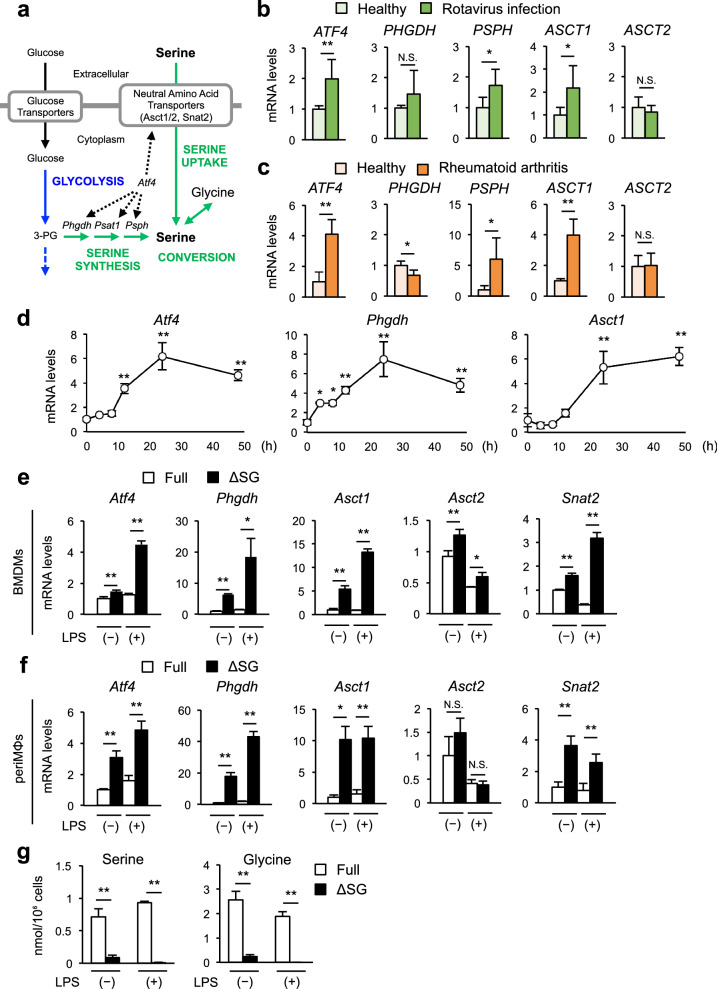
Macrophages under inflammatory conditions require exogenous serine to maintain intracellular serine levels. (**a**) Schema of serine metabolism. Neutral amino acid transporters include ASCT1, ASCT2, and SNAT2. (**b**) Gene expression of transcription factor and enzymes for serine biosynthesis and neutral amino acid transporters in peripheral blood mononuclear cells (PBMC) of 10 patients infected with rotavirus compared to 8 age-matched healthy controls (GEO dataset: GSE2729). (**c**) Gene expression of transcription factor and enzymes for serine biosynthesis and neutral amino acid transporters in macrophages from synovial fluids of 5 patients with rheumatoid arthritis compared to PBMC of 3 healthy donors (GEO dataset: GSE10500). (**d**) Gene expression of *Atf4*, *Phgdh* and *Asct1* in bone marrow-derived macrophages (BMDMs) at before and 4, 8, 12, 24, and 48 h after LPS stimulation. BMDMs were cultured in Full medium for 24 h, followed by stimulation with LPS (100 ng/ml) for the indicated times (n = 4). (**e,f**) Gene expression of *Atf4*, *Phgdh*, and neutral amino acid transporters in BMDMs (**e**) and peritoneal macrophages (periMΦs) (**f**). BMDMs or periMΦs were cultured in control (Full) or serine/glycine-depleted (ΔSG) medium for 24 h, followed by stimulation with LPS (100 ng/ml) for 8 h (n = 3). (**g**) Cellular content of serine and glycine in periMΦs analyzed by CE-TOF MS. PeriMΦs were cultured in Full or ΔSG medium for 24 h, followed by stimulation with LPS (100 ng/ml) for 6 h (n = 3). Values are means ± 95% CI. Statistical analysis was performed with unpaired *t* test (**b**,**c**,**e**–**g**) and Dunnett’s test (compared to 0 h (**d**)). *p < 0.05; **p < 0.01. See also Supplementary Figs. S1 and S2.

In most cell types, there are three major pathways to acquire serine, namely de novo serine synthesis, extracellular serine uptake, and conversion from glycine (Fig. 1a)^8,12^. Because serine is considered to be produced mainly within each cell and the role of serine uptake is largely unknown, we first analyzed the expression of representative genes involved in serine metabolism, such as activating transcription factor 4 (*ATF4*, a transcription factor that promotes serine metabolism genes), phosphoglycerate dehydrogenase (*PHGDH*, a rate-limiting enzyme involved in serine biosynthesis), phosphoserine phosphatase (*PSPH*, a key enzyme involved in serine biogenesis), and neutral amino acid transporters, including alanine serine cysteine transporters (*ASCT1* and *ASCT2*), using publicly accessible gene expression omnibus (GEO) datasets. As a result, expression of *ATF4, PSPH*, and *ASCT1* was upregulated in peripheral blood mononuclear cells of patients infected with rotavirus (Fig. 1b)^21^ and in synovial macrophages in patients with rheumatoid arthritis (Fig. 1c)^22^ compared to their respective healthy controls. Overall, genes involved in serine metabolism were upregulated by lipopolysaccharide (LPS) stimulation in mouse bone marrow–derived macrophages (BMDMs) as well (Fig. 1d), whereas the gene expression pattern of PHGDH was distinct between the species (Fig. 1b–d), probably due to its complex regulatory mechanism^23^. These observations led us to speculate that the demand for serine increases in immune cells under inflammatory conditions.

We then cultured BMDMs and peritoneal macrophages (periMΦs) in serine- and glycine-depleted medium (∆SG medium) and stimulated with LPS. In this study, we used ∆SG medium so that serine would not be provided by the conversion of glycine to serine (Fig. 1a). The expression of *Atf4*, *Phgdh* and neutral amino acid transporters (*Asct1*, *Asct2*, and system A amino acid transporter 2 (*Snat2*)) was significantly increased in macrophages, when they were cultured in ∆SG medium compared with the control medium containing all amino acids (Full medium), and expression of some genes was further upregulated by LPS stimulation (Fig. 1e,f). Despite the increased expression of these genes, cellular serine and glycine were almost exhausted in periMΦs (Fig. 1g, Supplementary Fig. S1) and BMDMs (Supplementary Fig. S2) cultured in ∆SG medium. These data indicate that deprivation of serine and glycine in culture media does not maintain their intracellular serine levels in macrophages during inflammation.

### Serine and glycine deprivation results in aberrant cytokine expression in macrophages

**Figure 2:**
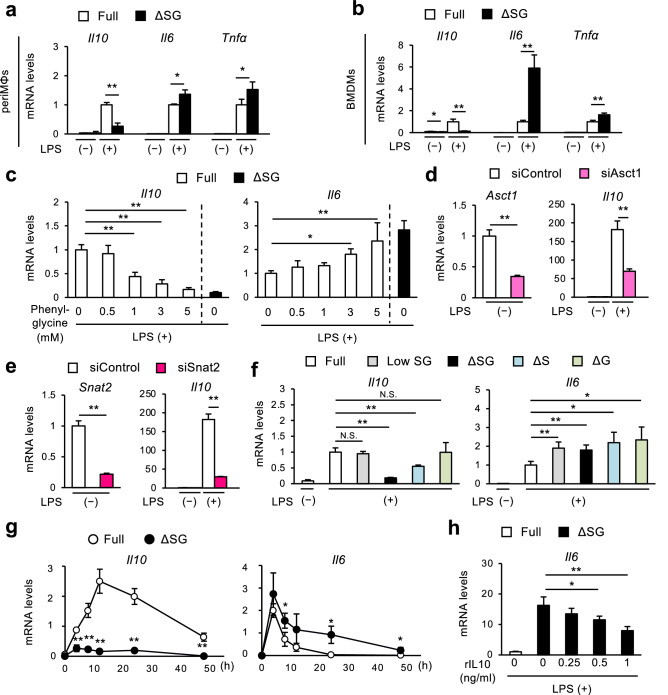
Serine and glycine deprivation results in aberrant cytokine expression in macrophages. (**a**,**b**) Gene expression of cytokines in periMΦs (**a**) and BMDMs (**b**). PeriMΦs and BMDMs were cultured in Full or ∆SG medium for 24 h, followed by stimulation with LPS (100 ng/ml) for 8 h and 24 h, respectively (n = 3–4). (**c**) Gene expression of *Il10* and *Il6* in BMDMs. BMDMs were cultured in Full or ∆SG medium with the indicated concentrations of 4-hydroxy-L-phenylglycine (an inhibitor of ASCT1 and ASCT2) for 24 h, followed by stimulation with LPS (100 ng/ml) for 8 h (n = 4). (**d**,**e**) Gene expression of *Asct1* and *Il10* (E) and of *Snat2* and *Il10* (F) in J774 macrophages transfected with siAsct1 (**e**), siSnat2 (**f**), and siControl were stimulated with LPS (100 ng/ml) for 24 h (n = 4). (**f**) Gene expression of *Il10* and *Il6* in BMDMs cultured in medium containing various levels of serine and glycine for 24 h, followed by stimulation with LPS (100 ng/ml) for 8 h. Full medium contains 400 µM serine and 400 µM glycine, Low SG medium contains 100 µM serine and 100 µM glycine, ∆SG medium contains neither serine nor glycine, ∆S medium contains no serine and 400 µM glycine, and ∆G medium contains 400 µM serine and no glycine, (n = 4). (**g**) Gene expression of *Il10* and *Il6* in BMDMs at before and 4, 8, 12, 24, and 48 h after LPS stimulation. BMDMs were cultured in Full or ∆SG medium for 24 h, followed by stimulation with LPS (100 ng/ml) for the indicated times (n = 4). (**h**) Gene expression of *Il6* in BMDMs. BMDMs were cultured in Full or ∆SG medium for 24 h, treated with the indicated concentrations of recombinant IL10 (rIL10) for 4 h, and then stimulated with LPS (100 ng/ml) for 24 h (n = 3). Values are means ± 95% CI. Statistical analysis was performed unpaired *t* test (**a**,**b**,**d**–**f**) and Dunnett’s test (**c**,**g**,**h**) (compared to Full + phenylglycine 0 mM (**c**); ∆SG + rIL10 0 ng/ml (**g**); Full + LPS ( +) (**h**)). *p < 0.05; **p < 0.01. See also Supplementary Figs. S3 and S4.

Next, we determined the effect of serine and glycine deprivation on cytokine expression in cultured macrophages (Fig. 2, Supplementary Fig. S3). In both periMΦs and BMDMs, treatment with LPS increased the expression of pro-inflammatory interleukin-6 (*Il6*) and tumor necrosis factor-α (*Tnfa*), together with anti-inflammatory *Il10* in Full medium (Fig. 2a,b, white bars). Depleting serine and glycine from the medium markedly suppressed LPS-induced IL10 expression and production, while significantly augmenting LPS-induced expression and production of inflammatory cytokines and chemokines including IL6 and TNFα (Fig. 2a,b, Supplementary Fig. S3a–c). ∆SG medium did not affect the expression of these genes without LPS treatment (Fig. 2a,b). Similar results were observed when BMDMs were stimulated with lower doses of LPS (Supplementary Fig. S3d).

Next, we treated BMDMs cultured in Full medium with 4-hydroxy-L-phenylglycine (phenylglycine), an inhibitor of ASCT1, to block the incorporation of extracellular serine to BMDMs. Phenylglycine suppressed LPS-induced expression and production of IL10 while augmenting those of IL6, both in a dose-dependent manner (Fig. 2c, Supplementary Fig. S3e). Similar results were obtained using siRNA for *Asct1* and *Snat2* (Fig. 2d,e). Because the selectivity of Asct1 and Snat2 for glycine is low, these findings suggest that extracellular serine is required to suppress LPS-induced aberrant cytokine production. To further distinct the role of serine and glycine, we cultured BMDMs in either medium containing lower concentrations (quarter amount of Full medium) of serine and glycine (Low SG medium), ∆SG medium, medium lacking only serine and not glycine (∆S medium), or medium lacking only glycine and not serine (∆G medium). Similar to ∆SG medium, ∆S medium significantly suppressed LPS-induced *Il10*, while Low SG or ∆G medium did not (Fig. 2f), indicating that extracellular serine but not glycine is required for *Il10* expression. On the contrary, all these media including ∆G medium significantly augmented LPS-induced *Il6* (Fig. 2f), suggesting that both serine and glycine regulate *Il6* expression. The distinct effects of glycine may be attributed to glutathione metabolism. As glycine is essential for glutathione synthesis (Supplementary Fig. S4a), the amount of glutathione was significantly reduced in periMΦs cultured in ∆SG medium compared with Full medium (Supplementary Fig. S4b). Consistently, glutathione replenishment reversed only *Il6* expression^24^, whereas it did not affect *Il10* expression (Supplementary Fig. S4c).

In addition, time course analysis revealed that suppression of *Il10* by ∆SG was observed as early as 4 h after LPS stimulation, whereas increase of *Il6* by ∆SG was evident only after 8 h and persisted up to 48 h (Fig. 2g). These observations led us to speculate that suppressed expression of *Il10* in macrophages cultured in ∆SG medium causes upregulation of *Il6* expression at the later time points, as previous studies reported that IL10 is crucial for resolution of inflammation^25^*.* Notably, IL10 supplementation reversed the otherwise increased *Il6* expression in ∆SG medium in a dose-dependent manner (Fig. 2h). Collectively, these findings suggest that suppressed *Il10* expression by deprivation of extracellular serine causes sustained inflammatory cytokine production. Thus, we sought to elucidate the molecular mechanism by which extracellular serine regulates *Il10* expression in cultured macrophages.

### Deprivation of serine and glycine alters cellular metabolism in macrophages

**Figure 3:**
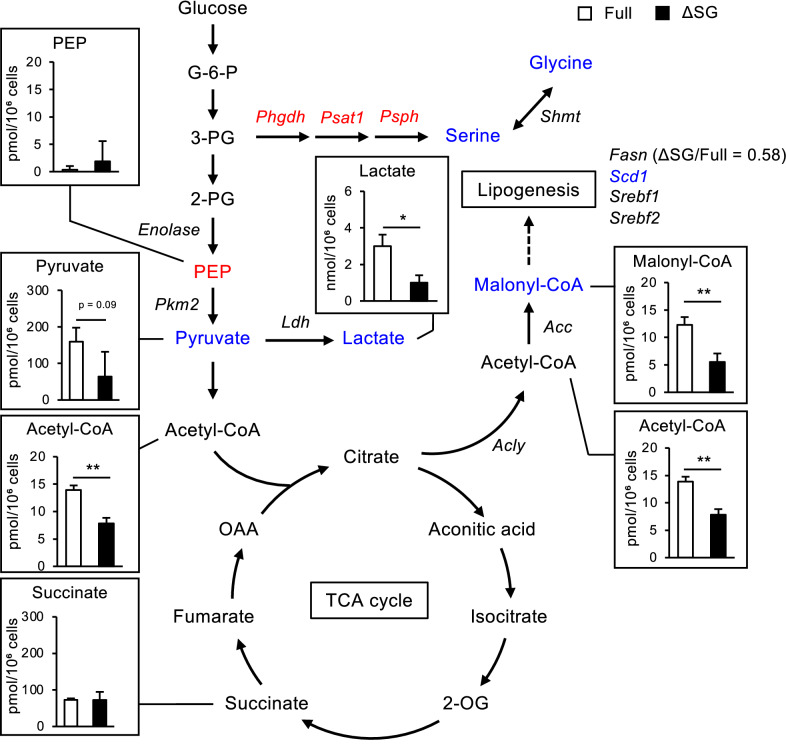
Serine and glycine deprivation alters cellular metabolism in macrophages. Transcriptomic and metabolic profiling in periMΦs cultured in Full or ∆SG medium for 24 h. Genes and metabolites whose expression and abundance were increased in ΔSG medium compared with Full medium (≥ 2.0) are highlighted in red, and those whose expression and abundance were decreased in ∆SG medium (≤ 0.5) are highlighted in blue. Gene names are indicated in italics. Values are means ± 95% CI. n = 3 and n = 4 for metabolomic and transcriptomic analyses, respectively. Statistical analysis was performed with Student’s *t* test. *p < 0.05; **p < 0.01. See also Supplementary Figs. S5 and S6.

To address the mechanism underlying *Il10* suppression by serine and glycine depletion, we performed transcriptomic and metabolomic profiling of LPS-stimulated periMΦs cultured in ∆SG or Full medium (Fig. 3, Supplementary Table S1). Metabolomic analysis revealed that depletion of serine and glycine from the culture medium markedly reduced their intracellular levels, along with acetyl-CoA, malonyl-CoA, lactate, and pyruvate, whereas the amount of phosphoenolpyruvic acid (PEP) tended to increase. The gap between the amount of PEP and pyruvate between the media is consistent with a previous report that serine is an allosteric activator of PKM2 (the M2 subtype of pyruvate kinase)^26^. The upregulation of genes related to de novo serine synthesis (*Phgdh*, phosphoserine aminotransferase 1 (*Psat1*), and *Psph*) was verified by transcriptome analysis, and reflected reduced intracellular serine levels. Moreover, there was a decrease in the amount of malonyl-CoA, a substrate for lipogenesis, and in the expression of stearoyl-CoA desaturase-1 (*Scd1*), a key enzyme in fatty acid metabolism. Thus, serine and glycine deprivation reduced the levels of several metabolites in the glycolysis and lipogenesis pathways. On the other hand, there was no apparent change in the cellular content of metabolites related to the tricarboxylic acid (TCA) cycle (Fig. 3, Supplementary Table S1), which may be due to compensation by anaplerotic pathways for the TCA cycle or a reduced TCA cycle flux. Taken together, these observations led us to determine the effect of several metabolites on aberrant cytokine expression in macrophages cultured in ∆SG medium. Replenishment of PEP, lactate, acetyl-CoA, and malonyl-CoA did not reverse the otherwise reduced expression of *Il10* in ∆SG medium (Supplementary Fig. S5). Moreover, deficiency of sterol regulatory element—binding transcription factor 1 (*Srebf1*), a key enzyme of de novo lipogenesis, in BMDMs did not affect *Il10* and *Il6* expression in ∆SG medium (Supplementary Fig. S6). Thus, we narrowed down the possible target molecules to just pyruvate.

### Reduction of mitochondrial pyruvate transport by serine and glycine deprivation is a cause of aberrant cytokine production

**Figure 4:**
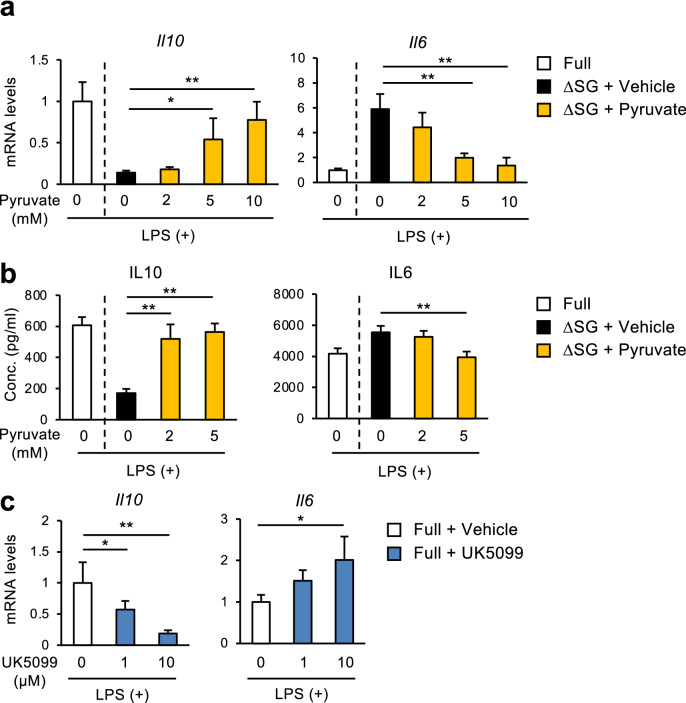
Reduction of mitochondrial pyruvate transport by serine and glycine deprivation is a cause of aberrant cytokine production. (**a**,** b**) Gene expression (**a**) and production (**b**) of cytokines in BMDMs. BMDMs were cultured in Full or ∆SG medium with indicated concentration of pyruvate for 24 h, followed by stimulation with LPS (100 ng/ml) for 24 h (n = 4). (**c**) Gene expression of *Il10* and *Il6* in BMDMs. BMDMs were cultured in Full with indicated concentration of UK5099 (an inhibitor of mitochondrial pyruvate carrier) for 24 h, followed by stimulation with LPS (100 ng/ml) for 24 h (n = 4). Values are means ± 95% CI. Statistical analysis was performed with one-way ANOVA Dunnett’s test (compared to ∆SG + Vehicle (**a**,**b**); Full + Vehicle (**c**)). *p < 0.05; **p < 0.01. See also Supplementary Figs. S5, S6, and S7.

Supplementation of pyruvate dose-dependently reversed the effect of serine and glycine deprivation on *Il10* and *Il6* expression in BMDMs (Fig. 4a). We verified these data by measuring of IL10 and IL6 concentrations in the media (Fig. 4b), suggesting the critical role of pyruvate in aberrant cytokine production in macrophages cultured in ∆SG medium. Pyruvate is imported into mitochondria via mitochondrial pyruvate carrier 1 (MPC1), and then oxidized to acetyl-CoA, which enters the TCA cycle (Fig. 3). Thus, we examined the effect of UK5099, an inhibitor of MPC1, on LPS-induced *Il10* and *Il6* expression in BMDMs cultured in Full medium. UK5099 treatment downregulated *Il10* expression while upregulating *Il6* expression, both in a dose-dependent manner (Fig. 4c). Although pyruvate replenishment and UK5099 treatment induced a modest increase in the concentrations of serine and glycine (Supplementary Fig. S7), it does not seem that these changes play a major role in regulating *Il10* expression. Collectively, these findings indicate that mitochondrial pyruvate transport plays a key role in regulation of cytokine expression in cultured macrophages.

### Impaired production of mitochondrial ROS is involved in aberrant cytokine expression under serine and glycine–depleted conditions

**Figure 5:**
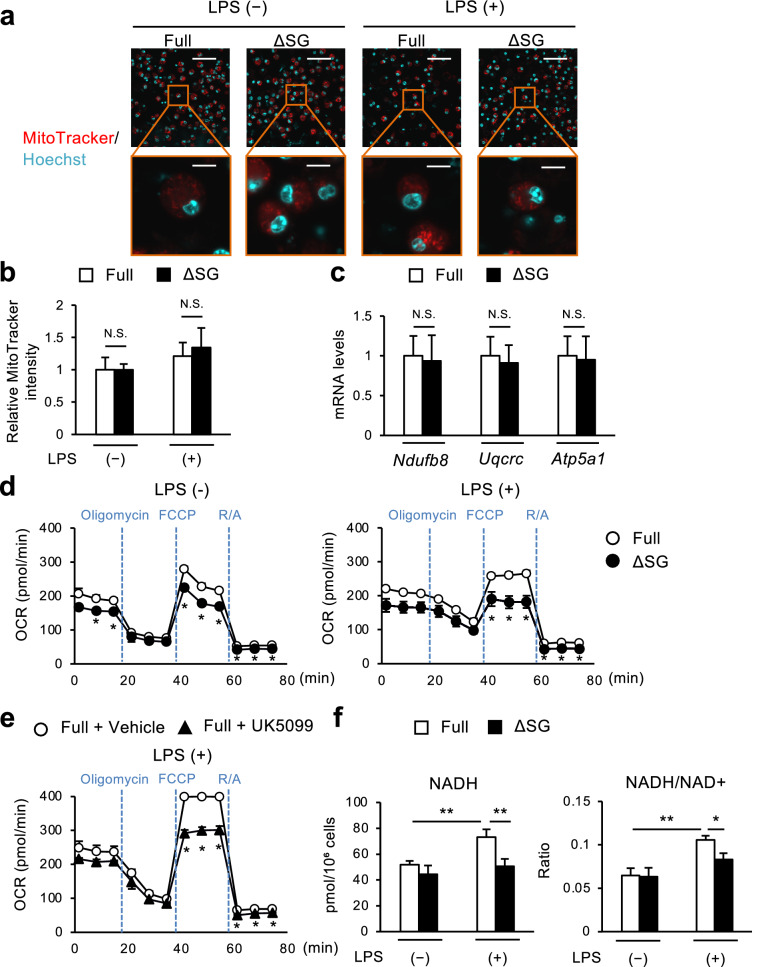
Deprivation of serine and glycine results in mitochondrial dysfunction in macrophages. (**a**,** b**) Representative images of mitochondria visualized by MitoTracker Red staining in BMDMs. Nuclei were counterstained with Hoechst33342. Scale bars, 50 μm (upper) and 300 µm (lower) (**a**). Flow cytometry analysis of mitochondrial mass in BMDMs by MitoTracker Red staining (**b**). BMDMs were cultured in Full or ∆SG medium for 24 h, followed by stimulation with LPS (100 ng/ml) for 8 h (n = 4). (**c**) Gene expression of mitochondrial OXPHOS complexes in BMDMs. *Ndufb8, Uqcrc* and *Atp5a1* are the genes of complex I, III and V, respectively. BMDMs were cultured in Full or ∆SG medium for 24 h (n = 3). (**d**) Oxygen consumption rate (OCR) of BMDMs. BMDMs were cultured in Full or ∆SG medium for 24 h followed by stimulation with vehicle (left) or LPS (100 ng/ml, right) for 6 h (n = 3). R/A: rotenone and antimycin. (**e**) OCR of BMDMs. BMDMs were cultured in Full or ∆SG medium with UK5099 (1 µM) for 24 h, followed by stimulation with LPS (100 ng/ml) for 6 h (n = 3). (**f**) Cellular content of NADH and NADH/NAD + in periMΦs. PeriMΦs were cultured in Full or ΔSG medium for 24 h, followed by stimulation with LPS (100 ng/ml) for 6 h (n = 3). Values are means ± 95% CI. Statistical analysis was performed with unpaired *t* test (**b**–**e**) with Bonferroni correction within treatments (0–20, 20–40, 40–60, 60–80 min) (**d**,**e**) and Dunnett’s test (**f**) (compared to Full + LPS). *p < 0.05; **p < 0.01; *NS* not significant.

Because mitochondria are multifunctional organelles, we sought to elucidate how serine and glycine deprivation influences mitochondrial functions in cultured macrophages. First, we visualized mitochondria using MitoTracker Red staining and found that ∆SG medium did not affect mitochondrial volume in BMDMs under both steady state and LPS-stimulated conditions (Fig. 5a,b). There was also no significant difference in expression of genes related to mitochondrial electron transport chain complexes between ∆SG and Full media (Fig. 5c). On the other hand, deprivation of serine and glycine significantly lowered the oxygen consumption rate (OCR) at steady state, and further lowered under LPS-stimulated conditions (Fig. 5d). These observations were recapitulated by UK5099 treatment in BMDMs cultured in Full medium (Fig. 5e). We also observed a significant reduction of the NADH content and the NADH/NAD^+^ ratio in macrophages cultured in ∆SG medium relative to Full medium (Fig. 5f). Collectively, these data suggest that serine deprivation decreases pyruvate transport into mitochondria and NADH levels, which results in reduction of OXPHOS.

**Figure 6:**
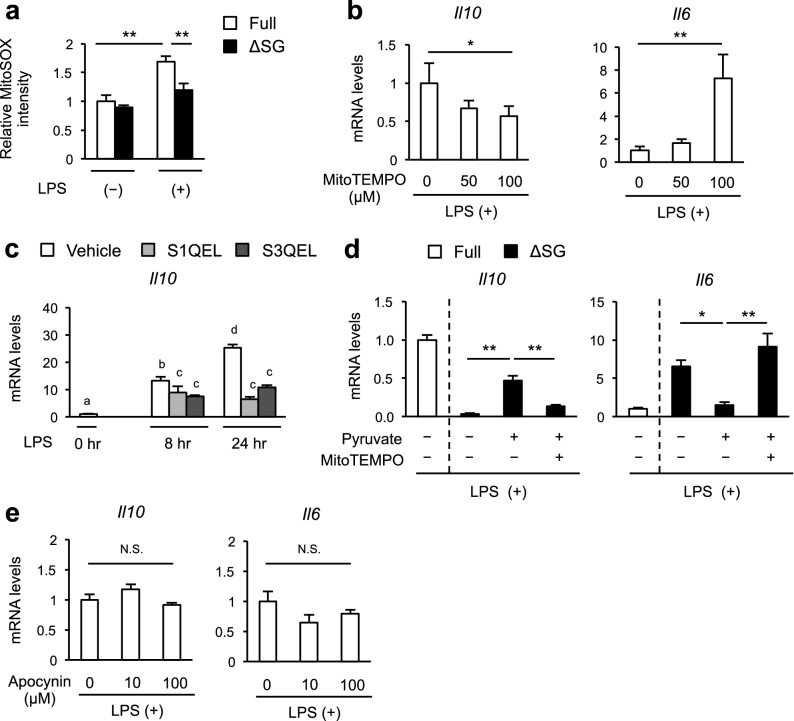
Impaired production of mitochondrial ROS by serine and glycine deprivation is a cause of aberrant cytokine expression. (**a**) Flow cytometry analysis of mitochondrial ROS production in BMDMs using MitoSOX (a mitochondrial superoxide indicator). BMDMs were cultured in Full or ∆SG medium for 24 h, followed by stimulation with LPS (100 ng/ml) for 8 h (n = 4). (**b**) Gene expression of *Il10* and *Il6* in BMDMs. BMDMs were cultured in Full medium with the indicated concentrations of MitoTEMPO (a mitochondria-targeted superoxide dismutase) for 24 h, followed by stimulation with LPS (100 ng/ml) for 24 h (n = 4). (**c**) Gene expression of *Il10* in BMDMs. BMDMs were cultured in Full medium for 24 h, treated with S1QEL or S3QEL (suppressors of complex 1 and 3, respectively) for 24 h, and then stimulated with LPS (100 ng/ml) for 8 h or 24 h (n = 4). (**d**) Gene expression of *Il10* and *Il6* in BMDMs. BMDMs were cultured in Full or ∆SG medium with pyruvate (10 mM) and/or MitoTEMPO (100 µM) for 24 h, followed by stimulation with LPS (100 ng/ml) for 24 h (n = 4). (**e**) Gene expression of *Il10* and *Il6* in BMDMs. BMDMs were cultured in Full medium for 24 h, treated with the indicated concentrations of apocynin for 1 h, then stimulated with LPS (100 ng/ml) for 8 h (n = 4). Values are means ± 95% CI. Statistical analysis was performed with Dunnett’s test (**a**, **b**, **d**, **e**) (compared to Full + LPS (**a**); Full + MitoTEMPO 0 µM (**b**); ∆SG + pyruvate + MitoTEMPO 0 µM (**d**)) and Tukey–Kramer’s test (**c**) (suffixed superscript letters differ significantly, *p* < 0.05). *p < 0.05; **p < 0.01; *NS* not significant.

Mitochondria is one of major sources of ROS. Although mitochondrial ROS (mROS) has been recognized as by-products of the TCA cycle and electron transport chain, recent evidence points to the role of mROS in various biological functions, including cytokine expression^27^. Using MitoSOX, a mitochondrial superoxide indicator, we found that LPS-induced mROS was significantly reduced in BMDMs cultured in ∆SG medium compared to Full medium, whereas there was no difference at steady state between the media (Fig. 6a). Blocking mROS using MitoTEMPO, a mitochondria-targeted antioxidant, effectively suppressed LPS-induced *Il10* expression while augmenting that of *Il6* (Fig. 6b). These data were confirmed using other chemicals suppressing mROS production through complex I and III (S1QEL and S3QEL, respectively) (Fig. 6c). Furthermore, MitoTEMPO treatment almost abolished the effect of pyruvate supplementation on *Il10* and *Il6* expression in ∆SG medium (Fig. 6d). In contrast, a specific NADPH-oxidase inhibitor apocynin to block ROS production mainly on plasma membrane but not in mitochondria, did not affect LPS-induced *Il10* and *Il6* expression in Full medium, suggesting that ROS differentially regulates cytokine expression depending on where it is produced (Fig. 6e). Collectively, these findings suggest that serine and glycine deprivation induces aberrant cytokine expression, at least partly, through impaired production of mROS in cultured macrophages (Supplementary Fig. S8).

## Discussion

Because inflammatory responses such as cytokine production, chemotaxis, and phagocytosis are energetically demanding processes, immune cells activate cellular metabolism to generate fuel under inflammatory conditions^28^. In this study, we have provided evidence that macrophages largely rely on exogenous serine to maintain the cellular serine levels during inflammation. This phenotype resembles that of neurons, which do not express key enzymes involved in de novo serine synthesis. Hence, surrounding glial cells supply serine to neurons via ASCT1^14^. However, unlike neurons, macrophages under serine deprivation upregulate the expression of genes related to serine metabolism. Why do macrophages require exogenous serine? It is probably attributed to the increased demand of serine under inflammatory conditions. Indeed, this study revealed that LPS stimulation induces the expression of genes related to de novo serine synthesis and neutral amino acid transporters in macrophages cultured in the Full medium. Consistently, monocytic cells of patients with a certain infectious disease or autoimmune disease exhibit similar expression patterns^21,22^, suggesting that de novo synthesis does not meet the high demand of serine. Accordingly, this study uncovered a fundamental mechanism, by which macrophages adapt to the nutritional microenvironment at the site of inflammation and modulate inflammatory cytokine production.

In this study, we used a combination of metabolomic and transcriptomic analyses to show that pyruvate, among a variety of other metabolites, is responsible for extracellular serine–regulated cytokine expression in macrophages. This notion is supported by several lines of evidence. For instance, Márquez et al*.*^29^ reported that inhibiting pyruvate transport to mitochondria by UK5099 suppresses IL10 expression in bone marrow–derived dendritic cells. Palsson-McDermott et al*.*^30^ showed that the activation of PKM2 or increased cellular pyruvate levels induce the expression of IL10 while suppressing IL1β in bone marrow–derived macrophages. Since serine is required for allosteric activation of PKM2, it is reasonable that cellular pyruvate levels are reduced under serine-depleted conditions^26^. In addition, serine racemase is supposed to produce pyruvate from serine^31,32^. Indeed, our transcriptomic data show that peritoneal macrophages express serine racemase at higher levels under serine-depleted conditions than at steady state (Kurita et al*.*, unpublished observations), which may be a compensatory mechanism for replenishing cellular pyruvate. Thus, pyruvate is a key metabolite for regulating inflammatory responses in immune cells, apart from its essential role in cellular energy metabolism.

Next, we must discuss the critical role of serine in one-carbon metabolism. Serine acts as a one-carbon donor, contributing to nucleotide synthesis, methylation reactions, and antioxidant production^8^. Substantial evidence has already shown that various cancer cells are dependent on serine in terms of cell proliferation and antioxidant defense, providing a novel therapeutic target for treating cancers^33,34^. Similarly, serine is required for T cell expansion in acquired immune response^10^. On the other hand, macrophages proliferate only under limited conditions in vivo, and little is known about the role of serine in non-proliferating cells. In this regard, several studies have reported that excessive serine administration can suppress inflammatory responses in certain animal models, by increasing glutathione synthesis^15–19^. Interestingly, our data indicate that glutathione replenishment does not affect *Il10* expression in macrophages, whereas we confirmed that glutathione supplementation effectively suppresses *Il6* expression. Nevertheless, we do not exclude the involvement of one-carbon metabolism in extracellular serine-regulated *Il10* expression, because Yu et al*.*^20^ recently reported that serine supports IL1β expression through S-adenosylmethionine production and histone methylation.

Amongst the myriads of cytokines, IL10 plays a critical role in resolving inflammation^25^. Macrophages are one of the major cellular sources of IL10 production, and also the main target cell type of the anti-inflammatory effects of IL10. Considerable attention has hitherto been paid to the pathophysiologic role of IL10 in various diseases. In terms of the mechanism of action, IL10 opposes the switch to the metabolic program induced by inflammatory stimuli^35^. However, the transcriptional regulation of IL10 remains incompletely understood. Using ROS inhibitors specific for mitochondria and NADPH oxidase, this study provides evidence that ROS production in mitochondria but not by NADPH oxidase is required for maximal expression of IL10 in LPS-treated macrophages. Consistently, mROS skews macrophage polarization toward an anti-inflammatory M2 phenotype^36^. Mills et al*.*^37^, however, have reported that mROS suppresses IL10 expression in macrophages. Therefore, further studies are needed to clarify the context-dependent effects of mROS on IL10 expression and the molecular mechanisms downstream of mROS.

In summary, we have demonstrated that macrophages require exogenous serine under inflammatory conditions to maintain their intracellular serine levels, in spite of increased expression of genes related to de novo serine synthesis and neutral amino acid transporters. Serine deprivation resulted in aberrant cytokine expression, particularly marked suppression of *Il10* expression, in macrophages. Our data also showed that the reduced pyruvate amount and its transport to mitochondria under serine deprivation suppress mROS, thereby inhibiting *Il10* expression. With regard to the clinical implications, similar metabolic changes may be observed in monocytic cells of patients with either viral infection or autoimmune disease^21,22^. Since the dose of LPS mainly used in this study is above the physiological levels, future studies are required to elucidate the metabolic changes that macrophages undergo in response to various inflammatory conditions in vivo. This study provides insight into novel molecular mechanisms regulating cytokine expression in macrophages, in response to the surrounding nutritional conditions.

## Methods

All methods were carried out in accordance with the ARRIVE guidelines.

### Resources and primers for Q-PCR

Information of key resources and primers for Q-PCR used in this study are shown in Supplementary Tables S2 and S3.

### Animals and cell culture

C57BL/6J mice were purchased from CLEA Japan or Japan SLC. All animals were housed in a temperature-, humidity- and light-controlled animal room (12 h light and 12 h dark cycle), and allowed free access to water and food (CE-2, CLEA Japan). Primary peritoneal macrophages (periMΦs) were obtained from 3% thioglycollate-treated mice 4 days after the injection. Murine bone marrow-derived macrophages (BMDMs) were obtained as described previously^38^. Briefly, bone marrow cells were isolated from femurs and tibias and differentiated in Iscove’s Modified Dulbecco’s Medium (Gibco) supplemented with 50 ng/mL recombinant human macrophage colony stimulating factor (M-CSF) (PeproTech) and 20% fetal bovine serum (FBS) for 6 days. J774 macrophages were cultured in Dulbecco’s Modified Eagle Medium (DMEM) (Nacalai Tesque) with 10% FBS.

### Gene expression and microarray analysis

Quantitative real-time PCR was performed as described^4^. In brief, total RNA was extracted from cells using Sepasol (Nacalai Tesque) and 10 ng of cDNA was used for real-time PCR amplification using SYBR Green Master mix (Thermo Fisher Scientific) and StepOne Plus instrument (Applied Biosystems). The primers used in this study are listed in Supplementary Table S3. Data were normalized to 36B4 and relative gene expression was calculated using comparative Ct method. For microarray analysis, RNA was purified using RNeasy MinElute Cleanup Kit, pooled from n = 4 biological replicates and processed at National Cerebral and Cardiovascular Center Hospital (Osaka, Japan) using Affymetrix GeneChip Mouse Genome 430 2.0 Arrays. Data was analyzed using Genespring GX (Agilent) and have been deposited in NCBI’s Gene Expression Omnibus (GSE156325, https://www.ncbi.nlm.nih.gov/geo/query/acc.cgi?acc=GSE156325).

### siRNA transfection

J774 macrophages were transfected with 50 nM siRNA targeted control GFP-22 (1022064, QIAGEN), *Asct1* (MSS225811, Thermo Fisher) or *Snat2* (MSS289807, Thermo Fisher) using Lipofectamine RNAiMAX (Thermo Fisher Scientific) according to the manufacturer’s instructions. Cells were incubated for 24 h prior to the assays. Knockdown of target genes was validated by real-time PCR.

### Cytokine production measurements

Cell-free supernatants were collected and the concentration of IL10 and IL6 in culture medium were measured by ELISA kits (R&D) according to manufacturer’s protocols.

### Oxygen consumption rate assays

Real-time oxygen consumption was measured using XFp Extracellular Flux analyzer (Agilent Technologies). BMDMs were seeded into Agilent Seahorse XFp Cell Culture Miniplate at a density of 1.0 × 10^5^ cells/well in Full or ∆SG medium and cultured overnight. Prior to the assay, cells were washed and incubated with XF Base Medium supplemented with 2 mM L-glutamine, 1 mM sodium pyruvate and 10 mM glucose at 37 °C without CO_2_ for 1 h. Seahorse XFp Cell Mito Stress Test were performed with 1 μM oligomycin, 1 μM FCCP and 0.5 μM Rotenone/antimycin A.

### Mitochondrial analysis

To stain mitochondria, the cells were incubated with 500 nM MitoTracker Red CM-H2Xros (Molecular Probes) in Full or ∆SG medium for 30 min at 37 °C. Cell nuclei were counterstained with Hoechst33342. Images were obtained using BZ-X710 fluorescent microscopy (KEYENCE). All images in the individual panels were acquired under room temperature with the same settings. To quantify the amount of mitochondria and mitochondrial ROS, cells were seeded in 96-well round-bottom plates at a density of 5 × 10^5^ cells/well and incubated with 500 nM MitoTracker Red CM-H2Xros (Molecular Probes) or 5 µM MitoSOX Red (Molecular Probes) in PBS with 1% BSA and 2 mM EDTA for 30 min at 37 °C to stain mitochondria and to examine the amount of mitochondrial ROS, respectively. After washing, mean fluorescence intensity was measured using MACSQuant Analyzer 10 (Miltenyi Biotec) and the data was analyzed with FlowJo V10 (BD Biosciences).

### Comprehensive metabolomic analysis.

Metabolomic analysis was performed at Human Metabolome Technologies (Tsuruoka, Japan). PeriMΦs (1.2 × 10^7^ cells) were seeded in 10 cm dish and cultured in DMEM medium containing 10% FBS for 4 h. Cells were then cultured in Full or ∆SG medium without FBS for 16 h, followed by stimulation with control PBS or 100 ng/ml LPS for 6 h. Cells were washed twice with 5% mannitol solution. Cellular metabolites were then extracted with 800 μl of methanol and 500 μl of distilled water containing HMT internal standard solution, as indicated by the manufacturer’s instruction. The extract was centrifuged at 2300×*g* for 5 min at 4 °C and the supernatant was filtrated using an ULTRAFREE MC-PLHCC 5 kDa-cutoff filter unit (HMT) by centrifugation at 9100×*g* for 35 min at 4 °C. Three biological replicates per condition were prepared. Metabolomic analysis was performed using CE-TOF MS/QqQ MS, and the metabolite peaks were quantified and normalized to the viable cell number.

### Amino acid analysis

Measurement of amino acids analysis in BMDMs was performed as described^39^. BMDMs (4 × 10^6^ cells) seeded in 6 cm dish were washed twice with PBS and collected with 250 µl of CH3OH, followed by addition of 10 μl of 0.5 mg/ml 2-isopropylmalic acid (Sigma-Aldrich) and10 μl of UL-13C,15 N-amino acid mixture (Taiyo Nissan, Tokyo, Japan) as internal standards dissolved in distilled water. The mixture was incubated for 30 min at 37 °C in a shaking incubator and then centrifuged at 16,000×*g* for 3 min at 4 °C. The supernatant was evaporated to dryness. As the first derivatizing agent, 40 μl of 20 mg/ml methoxyamine hydrochloride (Sigma-Aldrich) dissolved in pyridine was added and incubated for 90 min at 30 °C in a shaking incubator. The second derivatizing agent, 20 μl of N-methyl-N-trimethylsilyl-trifluoroacetamide (GL Science, Tokyo, Japan), was mixed and incubated for 30 min at 37 °C in a shaking incubator. The mixture was then centrifuged at 16,000×*g* for 3 min at 4 °C, and the resultant supernatant was transferred to a vial for gas chromatography/mass spectrometry (GC/MS) measurement. GC/MS was carried out using GCMS-TQ8040 (Shimadzu Co., Kyoto, Japan) with a DB-5 capillary column (30 m × 0.25 mm i.d.; 1 μm film thickness; Agilent J & W Scientific, Folsom, CA, USA). The inlet temperature was set at 250 °C, and the injection volume was 1 μL (splitless mode). The GC column temperature was programmed to remain at 100 °C for 4 min, followed by a 10 °C min^−1^ linear ramp to a final temperature of 320 °C, which was held for 11 min. Helium was used as a carrier gas at a flow rate of 1.1 mL min^−1^. The transfer line and ion source temperatures were maintained at 280 °C and 200 °C, respectively. For ionization, the electron impact mode at 70 eV was used. Argon gas was used as a collision-induced dissociation gas. Metabolites were detected using the Smart Metabolites Database (Shimadzu), which included the relevant multiple reaction monitoring (MRM) method file and data regarding the GC analytical conditions, MRM parameters, and retention index used for amino acid measurement following the report. The detected values were quantified and normalized to the viable cell number.

### Quantification and statistical analysis

All experiments were done at least three times independently. Data were analyzed using Prism Version 6 software (GraphPad). For data with two groups, unpaired t-tests were performed if homogeneity of variance and normality were confirmed, and by Welch’s test if not confirmed. The data were analyzed by one-way analysis of variance (ANOVA) followed by the post hoc Tukey–Kramer’s multiple comparison test for the comparison among 3 or more groups. When the values were compared only with the control values, Dunnett’s multiple comparison tests were used instead of Tukey–Kramer’s test. The time dependent changes were analyzed by repeated measures analysis of variance (ANOVA) with Bonferroni correction for comparisons between multiple groups and unpaired t-test for comparisons between two groups. Differences were assessed with two-side test with an α level of 0.05 and 0.01. Data are presented as the mean ± 95% Confidence Interval (CI). *p* < 0.05 was considered statistically significant.

### Study approval

All animal experiments were conducted in accordance with the guidelines for the care and use of laboratory animals of Nagoya University. The protocols were approved by the Animal Care and Use Committee, Research Institute of Environmental Medicine, Nagoya University (Approval Number 20253).

## Supplementary Information

Supplementary Information.

## Data Availability

The dataset generated in this publication have been deposited in NCBI’s Gene Expression Omnibus and are accessible through GEO: GSE156325 (https://www.ncbi.nlm.nih.gov/geo/query/acc.cgi?acc=GSE156325). Further information and requests for resources and reagents should be directed to and will be fulfilled by the Lead Contact, Takayoshi Suganami (suganami@riem.nagoya-u.ac.jp) or Ayaka Ito (aito@riem.nagoya-u.ac.jp).
