# Supplementary material for: Macrophages rely on extracellular serine to suppress aberrant cytokine production
Source: Sci Rep. 2021 May 27;11:11137. doi: 10.1038/s41598-021-90086-w (PMC8160356; doi:10.1038/s41598-021-90086-w)
Supplement: Supplementary file 1 — Supplementary Information. [file 41598_2021_90086_MOESM1_ESM.pdf]

Supplementary Information

Macrophages rely on extracellular serine to suppress aberrant cytokine production

Kento Kurita[1,2†], Hiroya Ohta[1,3, 9†], Ibuki Shirakawa[1,3†], Miyako Tanaka[1,3], Yasuyuki Kitaura[4], Yorihiro Iwasaki[5], Takashi Matsuzaka[6], Hitoshi Shimano[6], Seiichiro Aoe[7], Hiroshi Arima[2], Yoshihiro Ogawa[1,8], Ayaka Ito[1,3]*, Takayoshi Suganami[1,3]*

[1] Department of Molecular Medicine and Metabolism, Research Institute of Environmental Medicine, Nagoya University, Nagoya, Aichi, Japan

[2] Department of Endocrinology and Diabetes, Nagoya University Graduate School of Medicine, Nagoya, Aichi, Japan

[3] Department of Immunometabolism, Nagoya University Graduate School of Medicine, Nagoya, Aichi, Japan

[4] Laboratory Nutritional Biochemistry, Department of Applied Biosciences, Graduate School of Bioagricultural Sciences, Nagoya University, Nagoya, Aichi, Japan

[5] Center for Diabetes and Endocrinology, The Tazuke Kofukai Medical Research Institute Kitano Hospital, Osaka, Osaka, Japan

[6] Department of Endocrinology and Metabolism, Faculty of Medicine, University of Tsukuba, Tsukuba, Ibaraki, Japan

[7] Department of Home Economics, Otsuma Women's University, Tokyo, Japan

[8] Department of Medicine and Bioregulatory Science, Graduate School of Medical Sciences, Kyushu University, Fukuoka, Fukuoka, Japan

[9] Department of Pharmacology, Faculty of Pharmaceutical Sciences, Tokushima Bunri University, Tokushima, Tokushima, Japan

*Corresponding authors:

Takayoshi Suganami, M.D., Ph.D.; Department of Molecular Medicine and Metabolism, Research Institute of Environmental Medicine, Nagoya University, Nagoya, Japan. Tel: +81-52-789-3881, Fax: +81-52-789-5047, E-mail: suganami@riem.nagoya-u.ac.jp

Ayaka Ito, Ph.D.; Department of Molecular Medicine and Metabolism, Research Institute of Environmental Medicine, Nagoya University, Nagoya, Japan. Tel: +81-52-789-3883, Fax: +81-52-789-5047, E-mail: aito@riem.nagoya-u.ac.jp

[†] Kento Kurita, Hiroya Ohta, and Ibuki Shirakawa contributed equally to this work.

Table S1. Cellular metabolites in macrophages

Compound name	$HMDB_1$ ID	Full Average	ΔSG Average	ΔSG/Full Fold change	*p*-Value
Glycine	HMDB00123	2560.933	198.872	0.078	0.004
Serine	HMDB00187 HMDB03406	715.341	78.786	0.110	0.005
N-Acetylglutamic acid	HMDB01138	5.343	1.219	0.228	0.283
Lactic acid	HMDB00190 HMDB01311	3006.492	1001.587	0.333	0.012
Malonyl CoA	HMDB01175	12.268	4.909	0.400	0.003
Dihydroxyacetone phosphate	HMDB01473	23.592	10.455	0.443	0.202
Pyruvic acid	HMDB00243	159.707	73.159	0.458	0.148
Glutathione (GSH)	HMDB00125	2359.359	1094.667	0.464	0.002
PRPP	HMDB00280	34.109	17.240	0.505	0.066
N-Carbamoylaspartic acid	HMDB00828	9.154	4.788	0.523	0.145
Total Pyr-related Amino Acids	-	6303.269	3319.154	0.527	0.103
Lactate/Pyruvate	-	19.235	10.241	0.532	0.069
Total Glutathione	-	3260.565	1808.197	0.555	0.008
Acetyl CoA	HMDB01206	13.896	7.816	0.562	0.001
NADP+	HMDB00217	76.598	43.167	0.564	0.314
CoA	HMDB01423	13.446	7.821	0.582	0.011
GSH/GSSG	-	5.231	3.076	0.588	0.039
IMP	HMDB00175	25.094	16.002	0.638	0.021
Adenosine	HMDB00050	5.241	3.432	0.655	-
2-Hydroxyglutaric acid	HMDB00606 HMDB00694	90.754	64.572	0.712	0.015
Malate/Asp	-	0.059	0.042	0.714	0.063
ADP-ribose	HMDB01178	24.222	17.586	0.726	0.395
Glutamic acid	HMDB00148 HMDB03339	15820.499	11791.523	0.745	0.108
GMP	HMDB01397	48.335	36.305	0.751	0.031
6-Phosphogluconic acid	HMDB01316	5.321	4.014	0.754	0.056
β-Ala	HMDB00056	720.881	561.348	0.779	0.097
UDP-glucose	HMDB00286	311.432	245.805	0.789	0.093
Glutathione (GSSG)	HMDB03337	450.603	356.765	0.792	0.999
Total Glu-related Amino Acids	-	22170.716	17678.615	0.797	0.678
Total Non-essential Amino Acids	-	32026.606	25563.055	0.798	0.659

cis-Aconitic acid	HMDB00072	29.968	24.136	0.805	0.002
Total Glucogenic Amino Acids	-	35908.980	29092.146	0.810	0.952
Citric acid	HMDB00094	1501.179	1220.718	0.813	0.007
Total Amino Acids	-	37024.858	30128.808	0.814	0.964
Succinic acid	HMDB00254	73.359	61.302	0.836	0.945
Ribose 5-phosphate	HMDB01548	5.018	4.195	0.836	0.802
Creatine	HMDB00064	2238.801	1903.592	0.850	0.702
NADH	HMDB01487	51.828	44.198	0.853	0.154
XMP	HMDB01554	1.058	0.905	0.856	0.264
Malic acid	HMDB00156<br>HMDB00744	233.752	202.486	0.866	0.928
Valine	HMDB00883	885.379	767.755	0.867	0.507
NAD+	HMDB00902	803.021	706.233	0.879	0.066
Cysteine	HMDB00574<br>HMDB03417	258.835	228.813	0.884	0.442
2-Phosphoglyceric acid	HMDB03391	4.369	3.884	0.889	0.183
HMG CoA	HMDB01375	2.744	2.444	0.891	0.116
Threonine	HMDB00167	1310.032	1170.262	0.893	0.562
GDP	HMDB01201	64.250	57.480	0.895	0.138
Total Succinyl CoA-related Amino Acids	-	1973.444	1771.797	0.898	0.486
ATP	HMDB00538	3828.060	3441.099	0.899	0.241
Total Guanylate	-	849.425	764.637	0.900	0.139
AMP	HMDB00045	168.829	151.996	0.900	0.069
Uric acid	HMDB00289	9.699	8.744	0.902	0.496
Folic acid	HMDB00121	1.041	0.940	0.903	0.439
Total Adenylate	-	4236.935	3839.490	0.906	0.117
Total BCAA	-	2489.684	2257.947	0.907	0.479
GTP	HMDB01273	736.840	670.851	0.910	0.349
NADPH	HMDB00221	46.798	42.627	0.911	0.746
Total Essential Amino Acids	-	4998.253	4565.754	0.913	0.481
S-Adenosylmethionine	HMDB01185	71.002	64.898	0.914	0.269
cAMP	HMDB00058	4.025	3.682	0.915	0.315
Lysine	HMDB00182<br>HMDB03405	349.644	320.315	0.916	0.454
Methionine	HMDB00696	249.995	230.197	0.921	0.479
Ornithine	HMDB00214<br>HMDB03374	109.002	100.418	0.921	0.465

Phosphocreatine	HMDB01511	1144.803	1055.069	0.922	0.418
Isoleucine	HMDB00172	838.070	773.844	0.923	0.467
Fischer's Ratio	-	2.751	2.542	0.924	0.808
Glutamine	HMDB00641<br>HMDB03423	6111.112	5659.904	0.926	0.533
Adenylosuccinic acid	HMDB00536	4.154	3.857	0.928	0.108
Total Ketogenic Amino Acids	-	4165.709	3871.300	0.929	0.472
Total Acetyl CoA-related Amino Acids	-	2058.374	1915.545	0.931	0.461
Leucine	HMDB00687	766.235	716.348	0.935	0.462
Arginine	HMDB00517<br>HMDB03416	63.549	60.247	0.948	0.447
Fructose 1,6-diphosphate	HMDB01058	96.188	91.223	0.948	0.085
Histidine	HMDB00177	175.556	166.940	0.951	0.464
NADH/NAD+	-	0.065	0.063	0.966	0.857
3-Phosphoglyceric acid	HMDB00807	37.793	36.621	0.969	0.406
2,3-Diphosphoglyceric acid	HMDB01294	4.622	4.511	0.976	0.075
Tyrosine	HMDB00158	478.386	470.439	0.983	0.437
Total Fumarate-related Amino Acids	-	797.303	785.494	0.985	0.434
Total Aromatic Amino Acids	-	901.728	890.532	0.988	0.432
Phenylalanine	HMDB00159	318.917	315.055	0.988	0.430
Adenylate Energy Charge	-	0.932	0.928	0.996	0.719
Tryptophan	HMDB00929	104.425	105.039	1.006	0.422
Guanylate Energy Charge	-	0.905	0.915	1.011	0.071
Asparagine	HMDB00168	684.003	696.050	1.018	0.402
ADP	HMDB01341	240.046	246.395	1.026	0.484
Glucose 6-phosphate	HMDB01401	15.120	15.951	1.055	0.840
Fructose 6-phosphate	HMDB00124	5.729	6.486	1.132	0.828
Alanine	HMDB00161<br>HMDB01310	1353.703	1537.383	1.136	0.232
Glycerol 3-phosphate	HMDB00126	96.919	111.259	1.148	0.153
Total Oxaloacetate-related Amino Acids	-	4664.248	5537.087	1.187	0.160
Aspartic acid	HMDB00191<br>HMDB06483	3980.245	4841.037	1.216	0.115
Glucose 1-phosphate	HMDB01586	5.721	7.502	1.311	-
G6P/R5P	-	2.963	4.085	1.379	0.646
NADPH/NADP+	-	0.618	0.974	1.576	0.487

Choline	HMDB00097	133.813	216.435	1.617	0.136
Isocitric acid	HMDB00193	8.751	15.462	1.767	0.272
Glycerol 3-phosphate/DHAP	-	4.341	12.654	2.915	0.240
Phosphoenolpyruvic acid	HMDB00263	1.037	5.637	5.435	-

HMDB: The human metabolome database

Metabolic profiling in periMΦs cultured in Full or ΔSG medium for 24 h (n = 3).

Statistical analysis was performed with unpaired t-test.

Table S2. Key resources

Reagent or resource	Source	Identifier
**Chemicals, peptides, and recombinant proteins**		
4-Hydroxy-L-phenylglycine	Sigma-Aldrich	Cat# 56160
Acetyl-CoA	Sigma-Aldrich	Cat# A2056
Apocynin	Tokyo Chemical Industry Co., Ltd.	Cat# H0261
Glutathione reduced ethyl ester	Merck	Cat# G1404
Hoechst33342	Thermo Fisher Scientific	Cat# H1399
LPS	Sigma-Aldrich-Aldrich	Cat# L4391
Malonyl-CoA	Sigma-Aldrich	Cat# M4263
Methyl L-(-)-Lactate	Tokyo Chemical Industry Co., Ltd.	Cat# L0163
Methyl Pyruvate	Tokyo Chemical Industry Co., Ltd.	Cat# P0580
MitoSOX Red	Thermo Fisher Scientific	Cat# M36008
MitoTEMPO	Sigma-Aldrich	Cat# SML0737
MitoTrackerRedCMH2 X ROS	Thermo Fisher Scientific	Cat# M7513
PEP (Phosphoenolpyruvic Acid Monopotassium Salt)	Wako	Cat# 164-14761
Recombinant Human M-CSF	Peprotech	Cat# 300-25
Recombinant Murine IL-10	Peprotech	Cat# 210-10
S1QEL1.1	Cayman	Cat# 20982
S3QEL-2	Cayman	Cat# 18556
UK5099	Merck	Cat# 5.04817.0001
Thioglycollate	BD Biosciences	Cat# 225640
Lipofectamine RNAiMAX Transfection Reagent	Thermo Fisher Scientific	Cat# 13778075
**Critical Commercial Assays**		
Fast SYBR Green Master Mix	Thermo Fisher Scientific	Cat# 4385612
Mouse IL-10 Quantikine ELISA Kit	R&D Systems	Cat# M1000B
Mouse IL-6 Quantikine ELISA Kit	R&D Systems	Cat# M6000B
Seahorse XFp Cell Mito Stress Test Kit	Agilent	Cat# 103010-100
**Deposited Data**		
Microarray database		GEO: GSE156325
**Experimental Models: Cell Lines**		
J774.1 cell line	RIKEN BioResource Center	RRID:CVCL_4770

Mouse: bone-marrow derived macrophages	This paper: Mus musculus C57Bl6J	N/A
**Experimental Models: Organisms/Strains**		
Mouse: C57Bl6J	CLEA Japan	N/A
**Oligonucleotides**		
siAsct1	Thermo Fisher Scientific	Cat# 1320001
siSnat2	Thermo Fisher Scientific	Cat# 1320001
GFP-22 siRNA	Qiagen	Cat# 1022064
All primers are available in Table S3	Hokkaido System Science Co.,Ltd.	
**Software and Algorithms**		
FlowJo software v.10	BD	RRID:SCR_008520
Seahorse XFe96 Software Wave Desktop	Agilent	RRID:SCR_014526
Prism v.6.0c	GraphPad	RRID:SCR_002798

Table S3. Primers for Q-PCR

Primers for Q-PCR used in murine samples		
Atf4	F	5'-CTATGGATGATGGCTTGGCC-3'
Atf4	R	5'-CCGGAAAAGGCATCCTCC-3'
Atp5a1	F	5'-TGGGCGTGTGTTAAGCATTG-3'
Atp5a1	R	5'-TTCCCAAACACGACAACTCC-3'
Ccl2	F	5'-CCACTCACCTGCTGCTACTCAT-3'
Ccl2	R	5'-TGGTGATCCTCTTGTAGCTCTCC-3'
Ccl3	F	5'-AACCAAGTCTTCTCAGCGCC-3'
Ccl3	R	5'-GGAATCTTCCGGCTGTAGGAG-3'
Il10	F	5'-GCGCTGTCATCGATTTCTCC-3'
Il10	R	5'-CACCTGCTCCACTGCCTTG-3'
Il12b	F	5'-ACATCTACCGAAGTCCAATGCA-3'
Il12b	R	5'-GGAATTGTAATAGCGATCCTGAGC-3'
Il1b	F	5'-CTGGTGTGTGACGTTCCCAT TA-3'
Il1b	R	5'-CCGACAGCACGAGGCTTT-3'
Il6	F	5'-CCAGAGATACAAAGAAATGATGG-3'
Il6	R	5'-ACTCCAGAAGACCAGAGGAAAT-3'
Ndufb8	F	5'-AACCGATCACAGCATGAGAGG-3'
Ndufb8	R	5'-TATCGGTTCACCCCAGTTCATC-3'
Phgdh	F	5'-GGCCACCCGAATGCAAT-3'
Phgdh	R	5'-CGGCGACTTCAGGAGAGATG-3'
Asct1(Slc1a4)	F	5'-GGGAACGTGACCAAAGAGAA-3'
Asct1(Slc1a4)	R	5'-GGCCTCATTGAAGGAATTGAAG-3'
Asct2(Slc1a5)	F	5'-TGCTTTCGGGACCTCTTCT-3'
Asct2(Slc1a5)	R	5'-CCCGTTTAGTTGTGCGATG-3'
Tnfα	F	5'-ACCCTCACACTCAGATCATCTTC-3'
Tnfα	R	5'-TGGTGGTTTGCTACGACGT-3'
Uqcrc	F	5'-TGGGCGTGTGTTAAGCATTG-3'
Uqcrc	R	5'-TTCCCAAACACGACAACTCC-3'

Figure S1

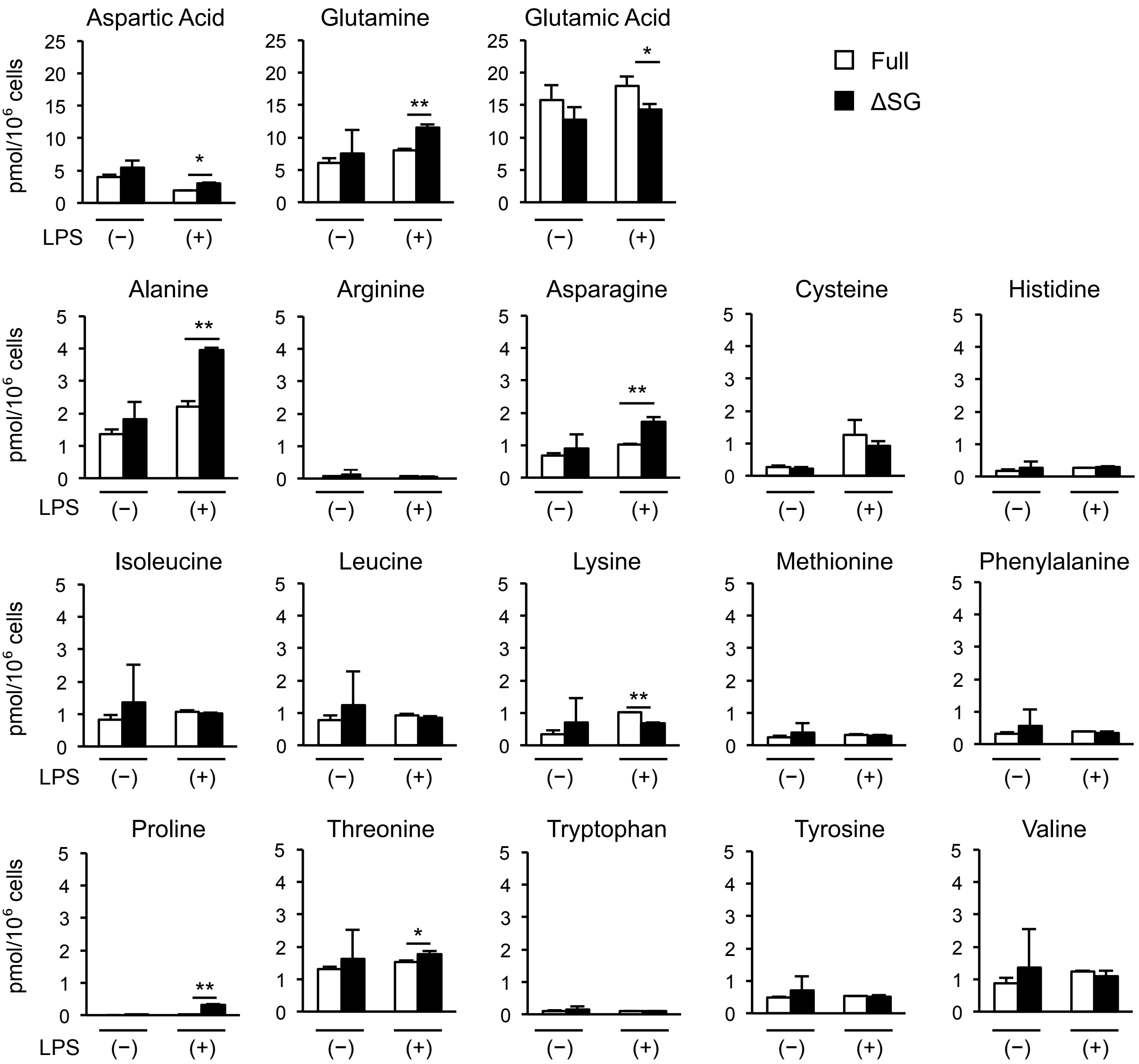

Figure S1. (Related to Fig. 1)
Effect of culture with the serine- and glycine-depleted medium on cellular amino acid content in peritoneal macrophages
Cellular content of amino acids in periMΦs analyzed by CE-TOF MS. PeriMΦs were cultured in Full or ΔSG medium for 24 h, followed by stimulation with LPS (100 ng/ml) for 6 h (n = 3). Cellular contents of serine and glycine are shown in Fig. 1g.
Values are means ± 95% CI. Statistical analysis was performed with unpaired t-test. *$p < 0.05$; **$p < 0.01$
Related to Fig. 1.

Figure S2

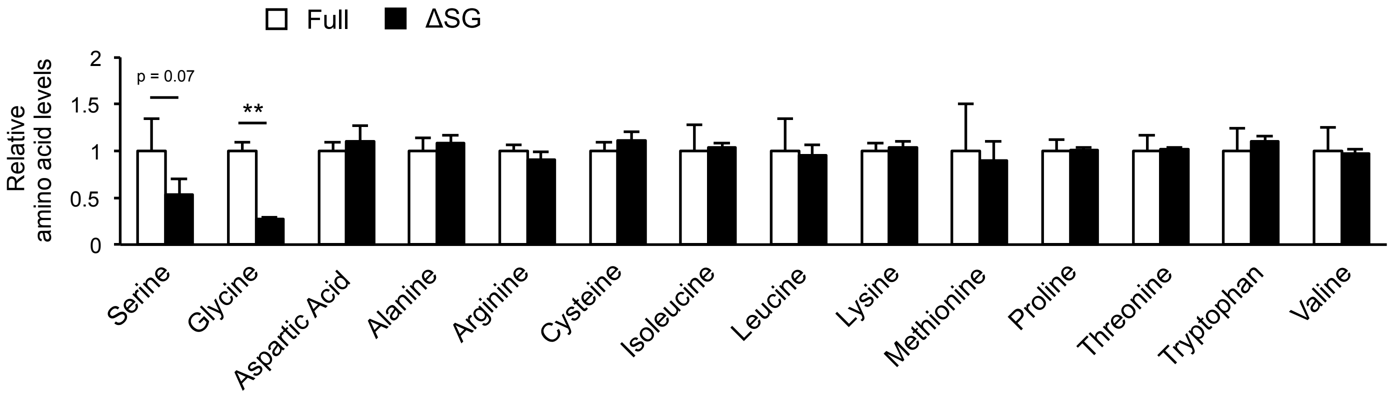

Figure S2. (Related to Fig. 1)
Effect of culture with the serine- and glycine-depleted medium on cellular amino acid content in bone marrow−derived macrophages
Relative cellular content of amino acids in BMDMs analyzed by GC/MS. BMDMs were cultured in Full or ΔSG for 24 h (n = 4).
Values are means ± 95% CI. Statistical analysis was performed with unpaired t-test. **p < 0.01. Related to Fig. 1.

Figure S3

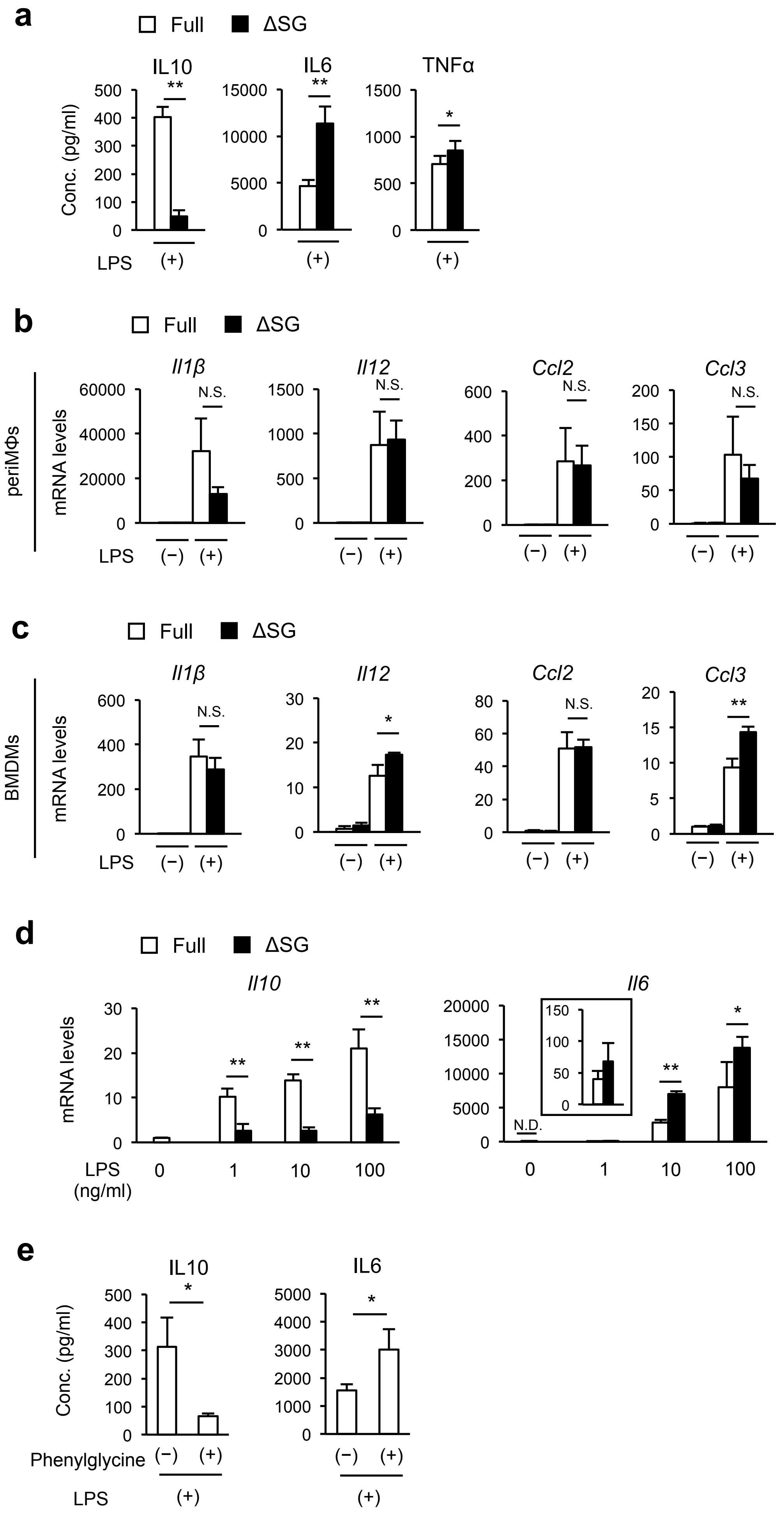
a
Full
ΔSG
IL10
IL6
TNFα
Conc. (pg/ml)
LPS
(+)
b
Il1β
Il12
Ccl2
Ccl3
periMΦs
mRNA levels
N.S.
(−)
(+)
c
BMDMs
d
Il10
Il6
N.D.
LPS (ng/ml)
0
1
10
100
e
Phenylglycine

Figure S3. (Related to Fig. 2)
Effect of serine and glycine deprivation on cytokine production in macrophages
(a) Production of cytokines in BMDMs. BMDMs were cultured in Full or ∆SG medium for 24 h, followed by stimulation with LPS (100 ng/ml) for 24 h (n = 4-6).
(b,c) Gene expression of cytokines in periMΦs **(c)** and BMDMs **(d)**. PeriMΦs and BMDMs were cultured in Full or ∆SG medium for 24 h, followed by stimulation with LPS (100 ng/ml) for 8 h and 24 h, respectively (n = 3–4).
(d) Gene expression of cytokines in BMDMs. BMDMs were cultured in Full or ∆SG medium for 24 h, followed by stimulation with LPS (1, 10 ng/ml) for 8 h (n = 4).
(e) Production of cytokines in BMDMs. BMDMs were cultured in Full medium with or without 4-hydroxy-L-phenylglycine (5mM, an inhibitor of ASCT1) for 24 h, followed by stimulation with LPS (100 ng/ml) for 8 h (n = 4).
Values are means ± 95% CI. N.D., not detected. Statistical analysis was performed with unpaired t-test. *p < 0.05; **p < 0.01; N.S., not significant. Related to Fig. 2.

Figure S4

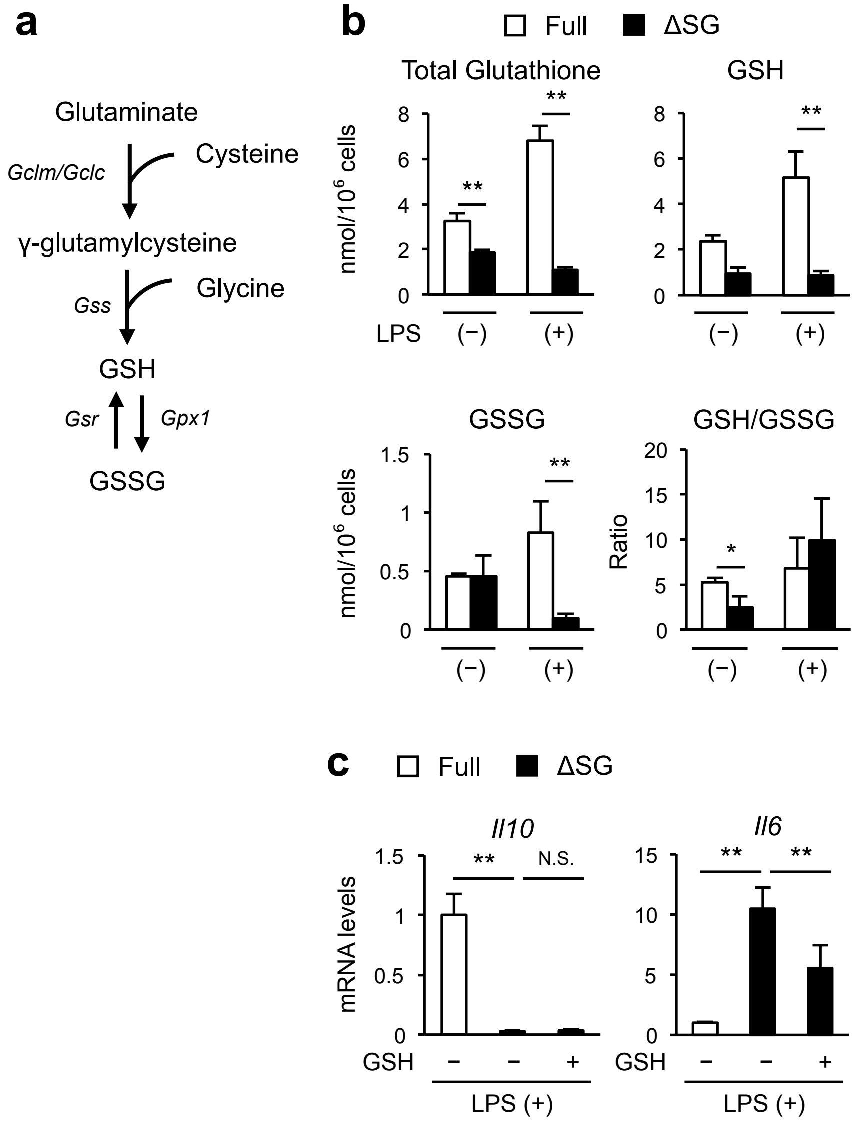

Figure S4. (Related to Fig. 2)

Serine and glycine deprivation results in impaired production of glutathione in macrophages

(a) Schema of glutathione metabolism.

(b) Cellular content of total glutathione, GSH, GSSG, and GSH/GSSG in periMΦs. PeriMΦs were cultured in Full or ΔSG medium for 24 h, followed by stimulation with LPS (100 ng/ml) for 6 h (n = 3).

(c) Gene expression of *Il10* and *Il6* in BMDMs. BMDMs were cultured in Full medium with GSH (1 mM) for 24 h, followed by stimulation with LPS (100 ng/ml) for 24 h (n = 4).

Values are means ± 95% CI. Statistical analysis was performed with unpaired t-test (b) and Dunnett's test (c) (compared to ΔSG + GSH (-)). *p < 0.05; **p < 0.01; N.S., not significant. Related to Fig. 2.

Figure S5

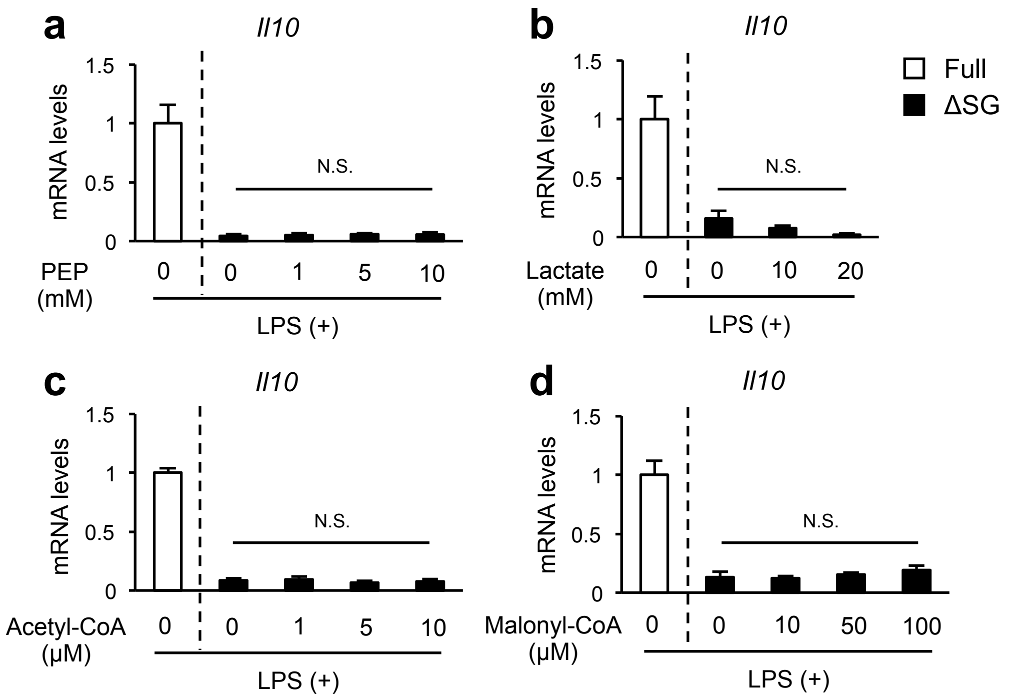

Figure S5. (Related to Figs. 3 and 4)
Replenishment of PEP, lactate, acetyl-CoA, or malonyl-CoA has little effect on the aberrant cytokine expression caused by serine and glycine deprivation
(a-d) Gene expression levels of *Il10* in BMDMs. BMDMs were cultured in Full or ΔSG medium with the indicated concentrations of phosphoenolpyrvate (PEP) (a), lactate (b), acetyl-CoA (c), or malonyl-CoA (d) for 24 h, followed by stimulation with LPS (100 ng/ml) for 24 h (n = 4).
Values are means ± 95% CI. Statistical analysis was performed with Dunnett's test (compared to ΔSG + PEP 0 mM (a); ΔSG + lactate 0 mM (b); ΔSG + acetyl-CoA 0 μM (c); ΔSG + malonyl-CoA 0 μM (d)). N.S., not significant. Related to Figs. 3 and 4.

Figure S6

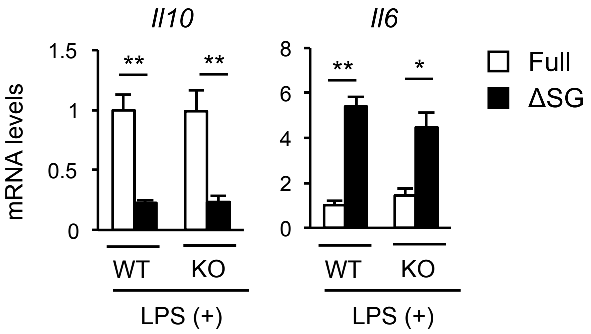

Figure S6. (Related to Figs. 3 and 4)
***Srebp1* deficiency does not affect cytokine expression caused by serine and glycine deprivation**
Gene expression of *Il10* and *Il6* in BMDMs obtained from wild-type (WT) or *Srebp1*-deficient (KO) mice. BMDMs were cultured in Full or ΔSG medium for 24 h, followed by stimulation with LPS (100 ng/ml) for 24 h (n = 4).
Values are means ± 95% CI. Statistical analysis was performed unpaired t-test. *p < 0.05; **p < 0.01 ; N.S., not significant. Related to Figs. 3 and 4.

Figure S7

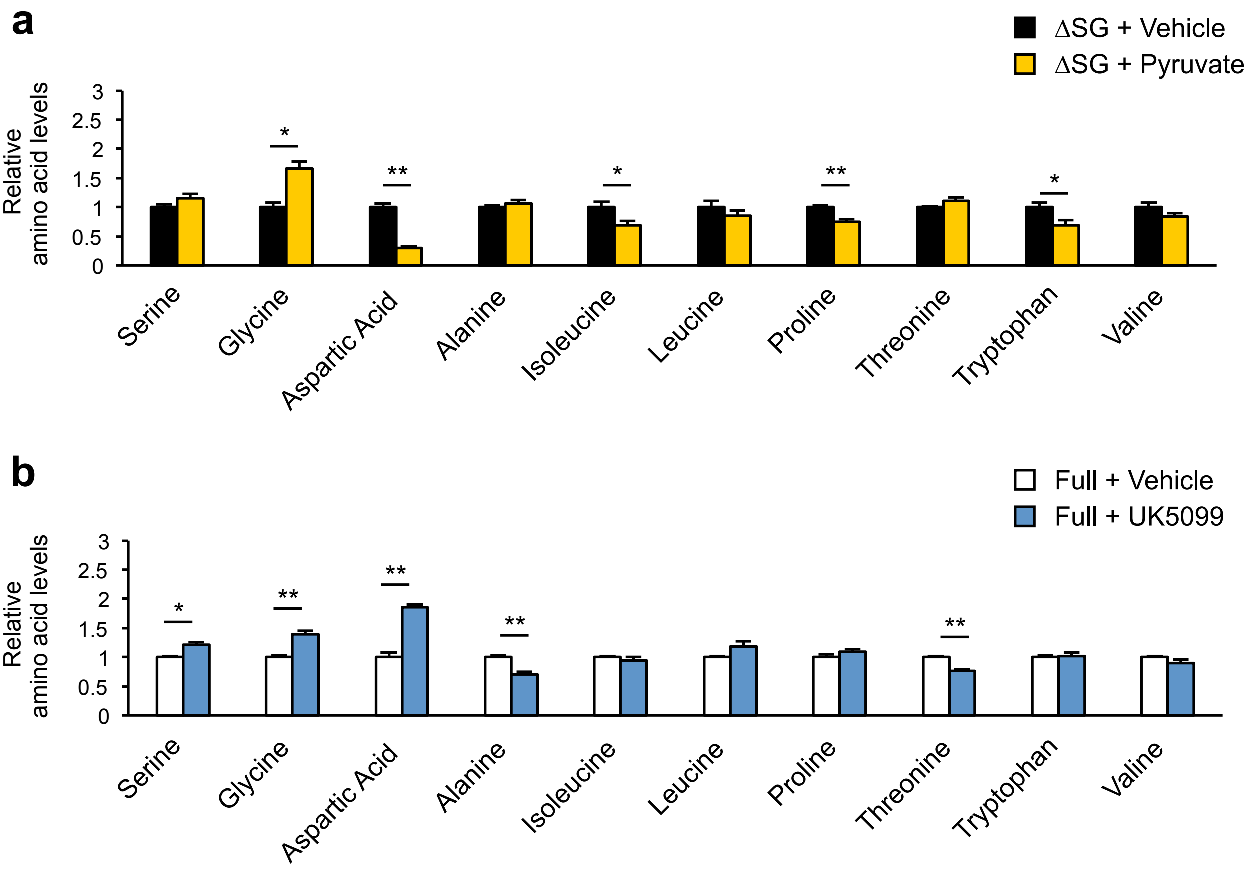

Figure S7. (Related to Fig. 4)
Effect of pyruvate treatment or inhibition of mitochondrial pyruvate transporter on cellular amino acid content in macrophages
(a) Relative cellular content of amino acids in BMDMs analyzed by GC/MS. BMDMs were cultured in ΔSG medium with or without pyruvate (10 mM) for 24 h (n = 4).
(b) Relative cellular content of amino acids in BMDMs analyzed by GC/MS. BMDMs were cultured in Full medium with or without UK5099 (10 μM, an inhibitor of mitochondrial pyruvate carrier) for 24 h (n = 4).
Values are means ± 95% CI. Statistical analysis was performed with unpaired t-test. *p < 0.05; **p < 0.01.
Related to Fig. 4.

Figure S8

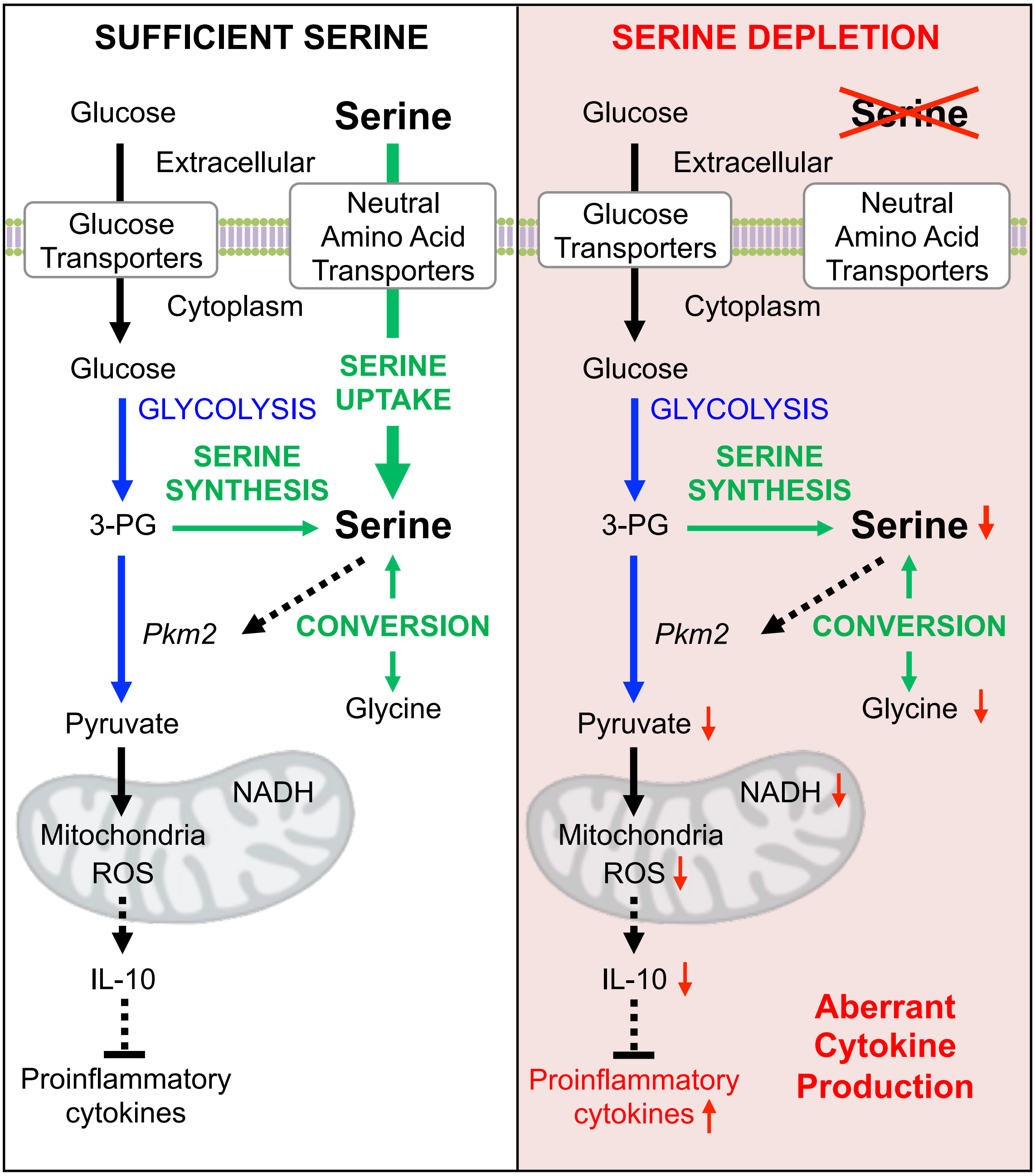

Figure S8.

Graphical summary: Potential molecular mechanisms underlying extracellular serine–regulated inflammatory cytokine production

In most cell types, there are 3 major pathways providing serine: *de novo* serine synthesis, uptake of extracellular serine, and conversion from glycine (*left*). However, the role of serine uptake has been largely unknown. Deletion of serine in the culture media remarkably reduced the cellular serine content in macrophages, indicating that macrophages largely depend on extracellular serine (*right*). Under serine deprivation, macrophages stimulated with LPS exhibited aberrant cytokine expression patterns, among which expression of anti-inflammatory *Il10* was markedly inhibited. In terms of the underlying mechanism, serine deprivation reduced pyruvate transport into mitochondria, impairing mitochondrial production of ROS. Supplementation of IL10 suppressed the sustained expression of *Il6*. Thus, this study demonstrates that macrophages rely on extracellular serine to suppress aberrant cytokine production.
